# Elevating Chest X-ray Image Super-Resolution with Residual Network Enhancement

**DOI:** 10.3390/jimaging10030064

**Published:** 2024-03-04

**Authors:** Anudari Khishigdelger, Ahmed Salem, Hyun-Soo Kang

**Affiliations:** 1 Department of Information and Communication Engineering, School of Electrical and Computer Engineering, Chungbuk National University, Cheongju 28644, Republic of Korea; khanudari1999@gmail.com; 2 Electrical Engineering Department, Faculty of Engineering, Assiut University, Assiut 71515, Egypt

**Keywords:** chest X-ray, super-resolution, residual network

## Abstract

Chest X-ray (CXR) imaging plays a pivotal role in diagnosing various pulmonary diseases, which account for a significant portion of the global mortality rate, as recognized by the World Health Organization (WHO). Medical practitioners routinely depend on CXR images to identify anomalies and make critical clinical decisions. Dramatic improvements in super-resolution (SR) have been achieved by applying deep learning techniques. However, some SR methods are very difficult to utilize due to their low-resolution inputs and features containing abundant low-frequency information, similar to the case of X-ray image super-resolution. In this paper, we introduce an advanced deep learning-based SR approach that incorporates the innovative residual-in-residual (RIR) structure to augment the diagnostic potential of CXR imaging. Specifically, we propose forming a light network consisting of residual groups built by residual blocks, with multiple skip connections to facilitate the efficient bypassing of abundant low-frequency information through multiple skip connections. This approach allows the main network to concentrate on learning high-frequency information. In addition, we adopted the dense feature fusion within residual groups and designed high parallel residual blocks for better feature extraction. Our proposed methods exhibit superior performance compared to existing state-of-the-art (SOTA) SR methods, delivering enhanced accuracy and notable visual improvements, as evidenced by our results.

## 1. Introduction

X-ray imaging captures internal body structures, portraying them in a grayscale spectrum where the tissue’s absorption of radiation dictates shades; notably, calcium rich bones, absorbing X-rays most prominently, manifest as bright white in the image [1,2,3,4]. Enhancing the pixel resolution of chest X-ray images is vital for sharpening image clarity, optimizing diagnostic precision, and identifying subtle abnormalities [5]. In recent years, the utilization of super-resolution (SR) has taken this enhancement further, refining image details and potentially unveiling nuanced features essential for precise medical assessments, offering a solution to improve the pixel resolution of medical images, including those produced through chest X-rays (CXRs), Magnetic Resonance Imaging (MRI), and Computerized Tomography (CT) [6]. SR aims to estimate high-resolution (HR) images from one or more low-resolution (LR) images, allowing for enhanced details and finer representation of image structures [5,6,7]. Furthermore, recent studies [5,6,8,9] show that SR can also help deep learning models to increase their segmentation performance. This technique has shown diverse applications, ranging from surveillance to medical imaging, offering potential advantages in medical image analysis [5,6,8,10,11].

In the field of SR, there are two primary approaches: Single Image Super-Resolution (SISR) and Multiple Image Super-Resolution (MISR) [5,10,12]. MISR is a computer vision technique that enhances the resolution and quality of an image by fusing information from multiple low-resolution input images. SISR focuses on reconstructing the HR output image from a single LR input image. Although both SISR and MISR methods have advantages, MISR is more challenging because of the difficulties in obtaining several LR images of the same object. SISR techniques have garnered acclaim for their elegant simplicity and remarkable efficacy in HR image reconstruction from a sole LR input [13,14]. CNN-based techniques have gained considerable traction in the SR field. Previous studies [5,8,14,15,16,17] underscore the benefits of leveraging SISR LR images to augment the effectiveness of deep learning models, encompassing GAN-based and Residual Group models in the SR field. However, it is crucial to recognize that GAN-based SR methods pose significant computational challenges. This is due to the intensive training requirements imposed by Generative Adversarial Networks (GANs), especially when dealing with high-resolution images. Additionally, the GAN-based methods with batch normalization behave differently during training and inference: they rely on batch statistics during training and population statistics during inference. These factors necessitate powerful hardware and substantial computational resources. 

In our novel approach, we embraced an Enhanced Residual network (i.e., a modified version of the RIR structure proposed by RCAN [8]) while eschewing the incorporation of a channel attention mechanism. This deliberate choice mitigates the computational load, particularly with HR images, rendering the process less computationally intensive and devoid of time-consuming aspects. Also, we incorporated the naive Inception architecture [18] into our proposed network to design parts of our proposed network (i.e., the naive Inception architecture was proposed for classification; it involves stacking multiple parallel convolutional pathways of different filter sizes and pooling operations to capture features at various scales within a single layer). In addition, we adopted dense feature fusion within our model for multi-stage information fusion. 

**Figure 1:**
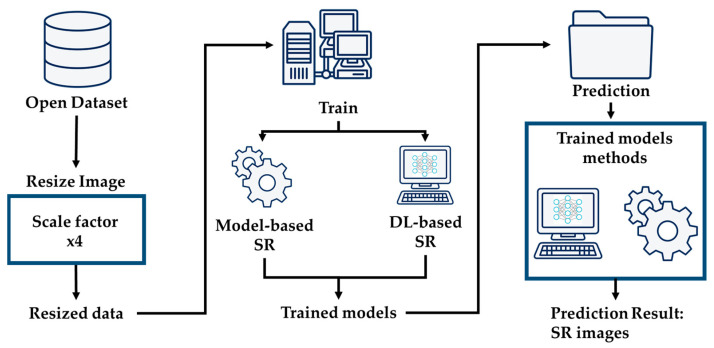
Structure of general system architecture.

**Figure 2:**
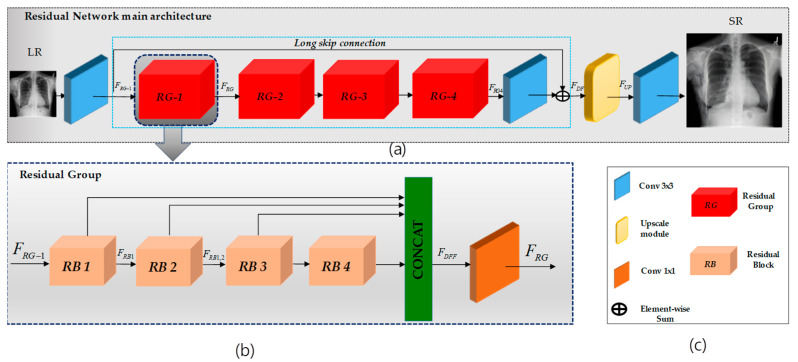
Overview of the proposed SR model. In (**a**), LR-to-HR transformation is depicted using convolutional layers within a residual-in-residual network with a long skip connection. The output is processed through an upsampling layer to generate the HR output image. (**b**) Each residual group comprises four residual blocks, incorporating multiplications such as 1 × 1, 3 × 3, and 5 × 5, depicted in the figure. Following these blocks, there is a 1 × 1 convolution. This process repeats for each residual group, and the outputs are concatenated before passing through a final 1 × 1 convolution layer. (**c**) expiation of the RN and RG architecture inside.

This research focuses on super-resolving chest X-ray images to enhance diagnostic precision. This enhancement provides physicians with detailed imagery for more precise analysis. Additionally, we explore cutting-edge super-resolution techniques, elucidating the overarching architectural framework depicted in Figure 1. We employ an advanced deep learning-based approach that utilizes residual learning to elevate the pixel resolution of CXR images, as depicted in Figure 2. This enhancement provides physicians with detailed imagery for more precise analysis. Additionally, we explore cutting-edge super-resolution techniques, elucidating the overarching architectural framework depicted in Figure 1. Furthermore, we apply bicubic downsampling by adopting the MATLAB function imresize from HR images with a scale factor of ×4. Subsequently, we add salt-and-pepper noise with noise levels of 0.005, 0.01, and 0.02 to each dataset.

The main contributions of this work can be summarized as follows:

- We harness the power of residual learning in medical CXR image SR, offering significant advancements in diagnostic precision and image quality.
- We adopted the RIR structure with dense feature fusion and highly parallel residual blocks comprising different kernel sizes, which enhances the diagnostic potential of CXR images. Our architecture incorporates four meticulously designed residual groups and blocks to extract and amplify spatial details. This facilitates the synthesis of HR CXR images, thereby advancing diagnostic imaging quality.
- Comprehensive experiments show that our proposed model yields superior SR results to the SOTA approaches.
- We conduct experiments involving salt-and-pepper noise, further demonstrating the robustness and effectiveness of our proposed approach in challenging imaging conditions.

## 2. Related Work

Recently, deep learning (DL)-based approaches to computer vision have dramatically outperformed traditional approaches. Single image super-resolution (SISR) and multiple image super-resolution (MISR) are the two broad categories into which the known SR techniques can be grouped [19,20]. This paper will primarily focus on SISR for medical X-ray images. By leveraging certain image priors, SISR algorithms aim to produce high-resolution (HR) images from low-resolution (LR) inputs.

### 2.1. Model-Based Super-Resolution Approaches

SISR algorithms can be categorized based on image priors. These algorithms include model-based methods, such as edge-based [21,22] models and image statistical models [20,23,24], patch-based methods [18,24,25], and learning-based approaches. Model-based approaches for super-resolution in medical imaging focus on incorporating prior knowledge or constraints of the image formation process to reconstruct high-resolution (HR) images from low-resolution (LR) inputs.

One common model-based technique is the Maximum Likelihood Estimation (MLE) framework [26]. MLE aims to maximize the likelihood of observing the LR image given the HR image and the degradation process. It models the degradation process, such as blurring and noise, to estimate the HR image that best explains the observed LR image.

Another widely used approach is the maximum a posteriori (MAP) estimation [22]. MAP incorporates prior information about the HR image, such as smoothness or sparsity, into the reconstruction process. MAP produces more accurate HR images by balancing data fidelity and prior information.

Regularization-based methods are also popular in model-based super-resolution [27]. These methods add a regularization term to the optimization problem to control the smoothness of the reconstructed HR image. The regularization term introduces constraints to achieve more plausible HR solutions.

### 2.2. Deep Learning-Based Super-Resolution Approaches

Deep learning-based SR methods employ neural networks to learn complex, nonlinear mappings that enhance image details effectively. These neural networks, often convolutional neural networks (CNNs), are trained using large datasets to grasp intricate relationships between LR and HR image patches, allowing for superior restoration of image details [8,14,16,17,24].

In the realm of super-resolution, various methods exhibit remarkable advancements. EDSR (Enhanced Deep Super-Resolution) [16] impresses with its highly accurate outcomes and exceptional image reconstruction quality, attributed to its efficient parallel architecture. This allows for swift processing—ideal for real-time applications—while maintaining parameter efficiency with fewer parameters than complex architectures. However, a notable drawback is EDSR’s demand for significant computational resources, especially for high up-scaling factors and large images, potentially hindering real-time usage on low- revise resource devices. On the other hand, VDSR (Very Deep Super-Resolution) [14] stands out due to Please its deep architecture, effectively capturing intricate image details and being less susceptible to overfitting, thereby aiding generalization to unseen data. Nevertheless, training deep models like VDSR poses challenges such as vanishing gradients, necessitating careful initialization and precise training strategies. Residual Dense Networks (RDN) [17] enhance information flow through skip connections and dense connectivity. Still, this advantage is balanced with increased model complexity and heightened memory usage, potentially limiting deployment on memory-constrained devices. These considerations highlight the trade-offs between efficiency and complexity in pursuing superior super-resolution methods. Shifting focus to Residual Channel Attention Networks (RCAN) [8], this innovative approach integrates an attention mechanism, enabling the model to prioritize critical features and significantly enhance reconstruction quality. RCAN introduced the residual-in-residual (RIR) structure, incorporating residual groups (RG) and long skip connections (LSC). Each RG comprises Residual Channel Attention Blocks (RCAB) with short skip connections (SSC). This innovative residual-in-residual architecture enables the training of very deep convolutional neural networks (CNNs) with over 400 blocks (i.e., the number of residual blocks in RCAN), significantly improving image super-resolution (SR) performance.

GAN-based super-resolution methods, such as SNSRGAN (Spectral-Normalizing Super-Resolution Generative Adversarial Network) [5], (i.e., using the same dataset and scale factor as our proposed model) and SRGAN (Super-Resolution Generative Adversarial Network) [28], showcase the immense potential of propelling image super-resolution, aiming to generate highly realistic high-resolution images. However, it is crucial to acknowledge and address two prominent challenges prevalent in these methods: Training Instability and Difficulty in Evaluation, which include the noteworthy impact of batch normalization (BN). BN utilizes batch statistics during training and population statistics during inference, potentially leading to inconsistencies and suboptimal results when transitioning from training to inference, consequently impacting the final quality of the generated high-resolution images. Training GANs for super-resolution introduces instability issues, often including problems like mode collapse, presenting significant obstacles to effective training and fine-tuning. Furthermore, accurately evaluating the performance of GAN-based SR methods remains a formidable task due to the absence of a well-defined objective metric, making the precise quantification of improvements a challenging endeavor.

This paper introduces a residual network that excludes batch normalization and channel attention, particularly tailored for X-ray image super-resolution. Notably, performing super-resolution on X-ray images adds more complexity than normal images (i.e., restoring fine details on X-ray images is very challenging). This study addresses this complexity by employing a deep learning-based super-resolution method incorporating a residual network (RN) specifically designed for chest X-ray images. We concentrate on chest X-ray images as our target data, aiming to overcome the intricacies of super-resolution effectively.

## 3. Methodology

This section will introduce the deep architecture and formulation of the proposed model using a convolutional residual network. The architecture of the model is shown in Figure 2.

### Network Overview

Our proposed network comprises three functional components, as illustrated in Figure 2. The initial segment of our network employs a single convolutional layer to extract shallow features, denoted as $N_{SF}$, from the low-resolution (LR) $I_{LR}$ input.

(1) $$N_{SF}=W_{SF}(I_{LR})$$

where $W_{SF}$ is the convolution layer’s shallow features. These shallow features are then used for deep feature extraction, denoted as $F_{DF}$, within the main network. The final functional component is the upscaling part, denoted as $F_{UP}$.

(2) $$I_{HR}=F_{UP}(F_{DF}+N_{SF})$$

**Figure 3:**
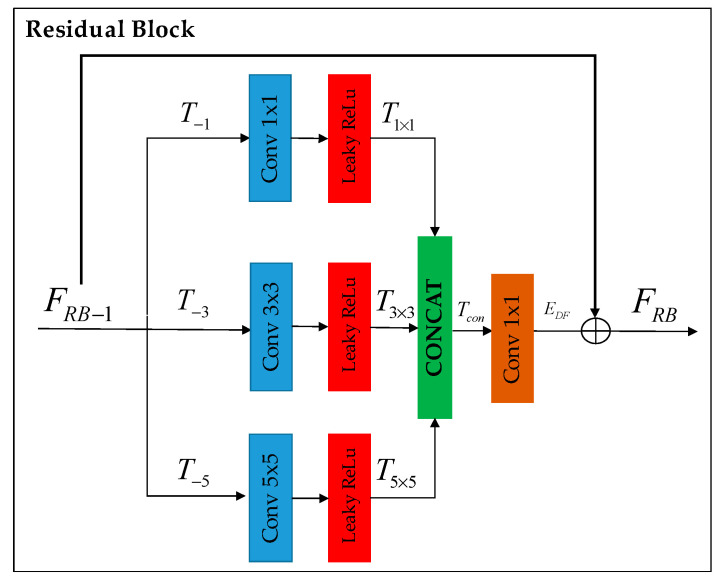
Residual block enhances deep feature extraction through parallel processing with different kernel sizes (i.e., 1 × 1, 3 × 3, and 5 × 5). The extracted features are then concatenated and fused to generate the residual block output. A short skip connection is used to concentrate more on extracting high-frequency details.

Deep feature extraction: Following RCAN [8], we adopted the residual-in-residual (RIR) structure, denoted as $N_{RIR}$, consisting of four residual groups (RG)—$F_{RG}$—and a long skip connection (LSC). Each RG further comprises four residual blocks (RB)—$F_{RB}$—(refer to Figure 3) involving a concatenation of three different kernel sizes along with a short skip connection (SSC).

(3) $$F_{DF}=N_{RIR}(F_{SF}(I_{LR}))$$

This residual-in-residual structure facilitates the training of a highly performant CNN with a deep structure for image SR. Remarkably, our approach is less time-consuming than that of the RCAN model while delivering superior results that surpass the performance achieved by the RCAN model (i.e., when using the same number of blocks). 

Residual Group (RG): Following RDN [17], we adopted dense feature fusion (DFF), denoted as $F_{DFF}$, within our RG to better utilize features extracted from the RBs hierarchically in a global way. Therefore, the features generated from the four RBs are concatenated along with the RG input and fused using a 1 × 1 convolution layer.

(4) $$F_{RG}=F_{DFF}(F_{RB1,}F_{RB2,}F_{RB3,}F_{RB4})$$

Residual Block (RB): Following Inception [18], we adopted the design of the naive version without using max pooling, as shown in Figure 3. The improvement of deep feature extraction within the residual block is achieved by simultaneously processing with various kernel sizes (namely, 1 × 1, 3 × 3, and 5 × 5), each followed by a *Leaky*Re*LU*.

T1×1=Conv1×1+LeakyReLu(T−1)

T3×3=Conv3×3LeakyReLu(T−3)

(5) T5×5=Conv5×5LeakyReLu(T−5)

The features obtained from this process are then merged and blended to create the output of the residual block.

(6) $$E_{DF}=T_{con}(T_{1\times 1},T_{3\times 3},T_{5\times 5})$$

where $E_{DF}$(**^.^**) and $T_{con}$(**^.^**) denote the RB in deep feature extraction and kernel size output concatenation. Additionally, a skip connection is incorporated to focus more on capturing high-frequency details.

(7) $$F_{RB}=F_{RB-a}+E_{DF}$$

(8) FRB=LeakyReLu(Fskip)

Upscaling Layer: The final extracted features (output of the RIR part) are then applied to the pixel shuffler layer to increase the spatial size [22]. Supposing the input to the pixel shuffler layer is the size of (H, W, C), the output generated is size (H/α, W/α, C/α^2^), where C represents the number of input channels and α represents the super-resolution factor (i.e., α = 4 in this article). Finally, the output of the pixel shuffler is applied to a convolutional layer with a kernel size of 3 × 3 to produce the final HR image.

## 4. Experiment

### 4.1. Datasets

This paper utilizes two chest X-ray datasets: Chest X-ray 14 [29] and Chest X-ray 2017 [30] and Chest X-ray 2017 comprises 5856 images from pediatric cases, with 4273 labeled as pneumonia (referred to as CXR 2) and 1583 as normal (referred to as CXR 3). We utilized the same dataset as SNSRGAN [5]. The dataset is split into training and testing sets, with 550 normal and 320 pneumonia images in the training set and 32 in the test set. The dataset characteristics and distribution are further illustrated in Table 1.

On the other hand, Chest X-ray 14, referred to as CXR1 in this paper, comprises 112,120 frontal-view chest X-ray images from 30,805 unique patients in the published NHCC American Research Hospital 2014 database [29]. Each image has a size of 1024 × 1024 pixels with 8-bit grayscale values [5]. Board-certified radiologists have annotated 880 bounding boxes for eight pathologies. For our analysis, we use the 32 annotated images as the testing set [5] and randomly select 250 images as the training set.

### 4.2. Implementation Details

All experiments were conducted on a 16-core Intel(R) Core(TM) i7-11700K processor with NVIDIA TITAN Xp 32 GB GPUs, ensuring consistency and objectivity. For the training dataset, we extracted accurate patches with sizes of 16 × 16 and 64 × 64 for input and ground-truth images, respectively, using a stride of one. This comprehensive dataset comprises 102,400 input and corresponding ground-truth patches, providing a substantial volume for robust training. Additionally, we utilized a batch size of 32 during the training process, and our implementation is based on high-level Python (TensorFlow).

### 4.3. Training Settings

We employ a bicubic kernel-based downsampling technique with a downsampling factor of $r=2^{k}$ (where k∈Z) to transform HR images into LR images, following the methodology outlined in SNSR-GAN. The model training process is optimized using the ADAM optimizer [31] with parameters β1 = 0.9, β2 = 0.999, and ε = 10^−8^. We initially set the learning rate to 2 × 10^−4^, with a subsequent exponential reduction by a factor of 0.1 every 120 epochs. Various loss functions are employed during the convolutional neural network (CNN) training, including L2 (sum of squared differences) and L1 (sum of absolute differences). As observed by Zhao et al. [5], L1 loss often outperforms L2 loss when assessing image quality using metrics such as Peak Signal-to-Noise Ratio (PSNR) and Structural Similarity Index (SSIM). In our study, we trained our proposed network to minimize the L1 distance between the original CXR input images and their corresponding ground-truth images [32].

In this paper, we compare the proposed method with the traditional interpolation methods, including nearest-neighbor (NN) [1] and bicubic interpolation [2], as well as several SOTA approaches, including SRCNN [15], VDSR [14], EDSR [16], RDB [17], SNSRGAN [5], and RCAN [8]. For fair comparison, we retrained on the same datasets we used to train our proposed model [29,30]. 

### 4.4. Evaluation Metrics

In image processing, quality assessment metrics are essential for evaluating the fidelity of reconstructed content. PSNR assesses quality by comparing the original and reconstructed signals, considering noise as interference. It quantifies the signal and noise power relationship, with higher PSNR values indicating superior quality, which is formulated as Equation (9). It is calculated based on the mean squared error (MSE) between the original and the processed images, considering the maximum possible pixel value (MAX), such as 255 for an 8-bit image. Higher PSNR values indicate better image quality [33].

(9) $$PSNR=10\times \log_{10}\left(\frac{MAX^{2}}{MSE}\right)$$

*SSIM* considers luminance, contrast, and structural information, reflecting pixel-wise similarity and preserving structural elements, making it a more perceptually meaningful metric. Formulated as Equation (10), it involves comparing images at multiple resolutions, capturing both fine and coarse details. The *SSIM* index is calculated based on the means ($\mu_{\chi }\mu_{y}$), variances ($\sigma_{x}^{2}+\sigma_{y}^{2}$), and covariance ($\sigma_{xy}$) of images *x* and *y.* Constants *C*_1_ and *C*_2_ are used to avoid instability when the denominator is close to zero. The *SSIM* value ranges from −1 to 1, where 1 indicates perfect similarity [34].

(10) $$SSIM(x,y)=\frac{\left(2\mu_{\chi }\mu_{y}+C_{1}\right)\left(2\sigma_{xy}+C_{2}\right)}{\left(\mu_{x}^{2}+\mu_{y}^{2}+C_{1})(\sigma_{x}^{2}+\sigma_{y}^{2}+C^{2}\right)}$$

Though less common, *MSIM* (multi-scale structure similarity index) is valuable as it captures average information loss during image compression, which is formulated as Equation (11) [34].

(11) $$MSIM={\left(I_{i=1}^{N}{\left(SSIM_{i}\right)}^{\alpha_{i}}\right)}^{\frac{1}{N}}$$

Lower *MSIM* values indicate superior compression quality, implying more faithful retention of structural information. These metrics collectively enable a robust evaluation of image processing methods, contributing to advancements in visual content enhancement and compression techniques. The specific formula for *MSIM* involves the product of the *SSIM* values at each scale, raised to the power of the corresponding weight ($\alpha_{i}$). The weighted product is then raised to the power of the reciprocal of the number of scales (*N*), providing a comprehensive multi-scale evaluation of structural similarity across different image detail levels. This paper complements qualitative assessments with quantitative measurements using *PSNR*, *SSIM*, and *MSIM*.

## 5. Results and Discussion

### 5.1. Comparisons with SOTA Methods

This research paper covers extensive experiments with deep learning-based SR methods. We are comparing our proposed model to these established approaches. These metrics offer a robust quantitative assessment. The objective was to enhance chest X-ray images’ visual quality and resolution, specifically those from three distinct datasets: CXR 1, CXR 2, and CXR 3. To evaluate the performance of these methods, we employed three key quality metrics—the PSNR, SSIM, and MSIM—to measure the fidelity and similarity between the super-resolved and ground-truth images. First, we set bicubic interpolation as a baseline and compared our efficiency against it; we achieved a PSNR improvement of 2.36 dB, 2.86 dB, and 4.47 dB on CXR 1, CXR 2, and CXR 3, respectively. In addition, visual results have shown that traditional interpolation methods excessively smooth out details, leading to noticeable results. In other words, these methods compromise fine details, which is unsuitable for medical images.

Subsequently, we compared our proposed model with SOTA deep learning-based SR techniques, including SRCNN, VDSR, EDSR, RDN SNSRGAN, and RCAN Table 2.

**Figure 4:**
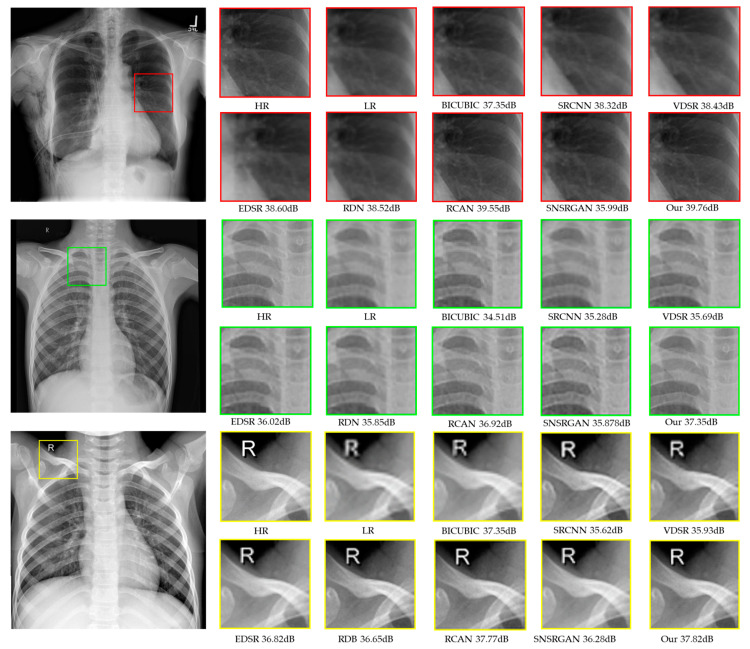
Visual comparison of super-resolved (up-scaled by a factor of 4×) chest X-ray images from the CXR1 (red squares), CXR2 (green squares), and CXR3 (yellow squares) datasets. The HR images generated through traditional interpolation methods, other SOTA methods, and our proposed model are presented.

The numerical results of different scale factors, illustrated in Table 2, present quantitative comparisons for ×4 SR. Among all previous methods, our proposed model consistently outperforms others across all datasets. Comparatively, SR methods relying on GANs produce more visually compelling results than other SOTA approaches. Nonetheless, a drawback arises regarding information loss when selectively enlarging areas of concern, as illustrated in Figure 4, which could impact diagnostic accuracy. Upon comparing the numerical results, we observed that RCAN performs the best after our proposed model. The enhancement in performance can be attributed to the additions and modifications incorporated in our model, such as dense feature fusion and highly parallel residual blocks.

Our study thoroughly explored diverse SR techniques, providing an analysis of their performance on various chest X-ray datasets. The comprehensive evaluation, which includes PSNR, SSIM, and MSIM metrics, highlights the potential of our proposed model as a robust solution for improving chest X-ray image quality. Additionally, we introduced noise at scale factors of ×2 and ×8 to assess the model’s performance under different conditions, further demonstrating its versatility and efficacy in enhancing chest X-ray images across various scenarios Table 2.

Furthermore, we conducted comparisons of datasets scaled by a factor of ×4, evaluating the performance of our proposed model against a GAN-based super-resolution model, specifically the SNSRGAN [5] model. While the SNSRGAN model demonstrated good performance on grayscale images, our proposed model surpasses its performance, as illustrated in Table 3.

### 5.2. Comparisons with SOTA Methods on Noisy Images

In this section, we delve into the experimental results concerning the efficacy of our proposed super-resolution model in enhancing noisy images, particularly those afflicted with salt-and-pepper noise—random isolated pixels of extreme brightness or darkness that distort the image [35]. Our investigation utilizes a scaling factor ×4 dataset comprising chest X-ray (CXR) images deliberately corrupted with varying noise levels (0.005, 0.01, and 0.02) to simulate different degrees of image distortion (Table 4). We compare our proposed model against several state-of-the-art super-resolution algorithms, including BICUBIC, SRCNN, VDSR, EDSR, RDN, RCAN, and SNSRGAN for noisy LR images.

Our experimental findings, meticulously outlined in Table 4, underscore the superior performance of our proposed model across various noise levels and CXR datasets (CXR1, CXR2, CXR3) on ×4 scaling factors. Notably, at a noise level of 0.005, our model consistently surpasses baseline methods, yielding substantial improvements in Peak Signal-to-Noise Ratio (PSNR) of 14.29% (CXR1), 12.5% (CXR2), and 12.5% (CXR3), alongside corresponding Structural Similarity Index (SSIM) values of 0.806, 0.818, and 0.801, respectively.

Further analysis reveals the intricate challenges posed by noise levels ranging from 0.01 to 0.02 in LR images, where traditional methods struggle to extract high-level noise details effectively. However, our proposed RN model demonstrates remarkable capabilities in managing such noise complexities, effectively suppressing noise and recovering additional image details in most scenarios. The detailed comparison provided in Table 4 highlights the significant advantage of our RN model over existing methods, particularly in its ability to handle diverse noise levels and enhance image quality. This comparison solidifies the efficacy of our proposed approach and suggests its potential for integration with complementary techniques such as super-resolution and image denoising.

### 5.3. Ablation Study

We evaluate six distinct architectures to illustrate the impact of various components in the model, as presented in Table 5. All models listed in Table 5 share the same residual groups and blocks, where RG, RB, and f denote the number of Residual Groups, Residual Blocks, and filters for each convolutional layer, respectively. Having constructed our model on the residual-in-residual structure, we conducted a comparison with RCAN. We assessed the impact of the modified residual block (specifically, the highly parallel residual block with varying kernel sizes). We examined the influence of incorporating the channel attention mechanism (CA), which we did not utilize. The first four rows of results shoFwcase our outcomes with and without the use of the CA; without the use of concatenation, with the CA; and the CA, while the fifth and sixth rows present the results of the RCAN model with and without the use of the CA.

The model performs better when not using the CA, contrary to what was suggested in RCAN. This discrepancy is reasonable, considering the distinct nature of the chest X-ray images. Furthermore, comparing the results in the second and fifth rows demonstrates that our proposed model exhibited exceptional performance across all datasets, surpassing the RCAN method in terms of PSNR.

Furthermore, in our study, we emphasize using skip connections and concatenation. Specifically, our model integrates concatenation with long skip connection within each residual block.

## 6. Conclusions

In this work, we introduced a learning-based super-resolution approach specifically tailored to enhance chest X-ray images. By harnessing the inherent strength of the residual-in-residual structure, we have meticulously designed our network to extract deep features effectively. Through the integration of dense feature fusion and the utilization of highly parallel residual blocks, we have further fortified the network’s capacity to comprehend and model intricate relationships within the images, consequently restoring finer texture details and enhancing overall quality. Moreover, through comparative analysis with noisy images using various super-resolution models, our findings indicate that our proposed model exhibits significant denoising capabilities for low-resolution images. Across diverse datasets, our proposed model has consistently demonstrated exceptional performance, outperforming existing methods in terms of Peak signal-to-noise ratio (PSNR). This highlights the remarkable capability of our model to enhance the quality of chest X-ray images, thereby positioning it as a robust solution for comprehensive image enhancement in medical imaging applications.

## Figures and Tables

**Table 1:** Dataset characteristics and distribution.

Scale	CXR1 [29]		CXR2 [30]		CXR3 [30]	
	Test	Train	Test	Train	Test	Train
×2	32	250	87	550	185	880
×4	32	250	87	550	185	880
×8	32	250	87	550	185	880

**Table 2:** Numerical comparison of super-resolved chest X-ray images, up-scaled by factors of 2×, 4×, and 8×. The PSNR, SSIM, and MSIM are presented with bolded values to indicate the best performance.

Scale	Methods	CXR1 [29]	CXR2 [30]	CXR3 [30]
		*PSNR/SSIM/MSIM*	*PSNR/SSIM/MSIM*	*PSNR/SSIM/MSIM*
X2	BICUBIC [24]	34.82 dB/0.787/0.86	32.42 dB/0.824/0.873	29.96 dB/0.797/0.875
	SRCNN [18]	35.52 dB/0.717/0.891	32.28 dB/0.829/0.929	30.17 dB/0.812/0.891
	VDSR [14]	35.62 dB/0.837/0.950	33.85 dB/0.871/0.936	33.83 dB/0.862/0.923
	EDSR [16]	35.80 dB/0.896/0.977	34.35 dB/0.873/0.935	33.92 dB/0.892/0.949
	RDN [17]	36.72 dB/0.915/0.962	34.65 dB/0.892/0.948	35.12 dB/0.902/0.961
	RCAN [8]	36.95 dB/0.926/0.972	35.02 dB/0.901/0.953	36.77 dB/0.908/0.963
	**PROPOSED**	**37.11 dB/0.936/0.9825**	**36.15 dB/0.912/0.968**	**37.89 dB/0.918/0.979**
X4	BICUBIC [24]	37.35 dB/0.907/0.940	34.51 dB/0.901/0.910	33.35 dB/0.907/0.910
	SRCNN [18]	38.32 dB/0.9392/0.941	35.28 dB/0.921/0.929	35.62 dB/0.912/0.932
	VDSR [14]	38.42 dB/0.938/0.940	35.69 dB/0.917/0.936	35.93 dB/0.92/0.938
	EDSR [16]	38.60 dB/0.944/0.967	36.02 dB/0.925/0.945	36.82 dB/0.932/0.959
	RDN [17]	38.52 dB/0.939/0.972	35.85 dB/0.912/0.968	36.22 dB/0.925/0.958
	RCAN [8]	39.55 dB/0.947/0.982	36.92 dB/0.927/0.972	37.77 dB/0.921/0.953
	**PROPOSED**	**39.76 dB/0.944/0.991**	**37.38 dB/0.932/0.989**	**37.82 dB/0.937/0.982**
X8	BICUBIC [24]	29.18 dB/0.773/0.820	28.21 dB/0.751/0.810	28.55 dB/0.767/0.820
	SRCNN [18]	29.32 dB/0.792/0.841	29.08 dB/0.781/0.839	29.62 dB/0.792/0.832
	VDSR [14]	30.62 dB/0.838/0.890	31.69 dB/0.841/0.896	30.13 dB/0.882/0.898
	EDSR [16]	30.91 dB/0.844/0.907	31.02 dB/0.849/0.902	30.62 dB/0.892/0.939
	RDN [17]	32.12 dB/0.849/0.912	32.85 dB/0.871/0.928	31.95 dB/0.872/0.928
	RCAN [8]	32.87 dB/0.859/0.932	32.92 dB/0.897/0.952	33.87 dB/0.881/0.9453
	**PROPOSED**	**33.17 dB/0.865/0.942**	**33.48 dB/0.912/0.968**	**34.40 dB/0.901/0.9625**

**Table 3:** Numerical comparison of super-resolved chest X-ray images, up-scaled by a factor of 4×. The PSNR, SSIM, and MSIM are presented with bolded values to indicate the best performance.

Methods	CXR 1 [29]	CXR2 [30]	CXR3 [30]
	*PSNR/SSIM/* *MSIM*	*PSNR/SSIM/* *MSIM*	*PSNR/SSIM/* *MSIM*
RCAN [8]	39.55 dB/0.947/0.982	36.92 dB/0.927/0.960	37.77 dB/0.931/0.953
SNSRGAN [5]	35.99 dB/0.924/0.983	35.87 dB/0.910/0.979	36.28 dB/0.915/0.943
PROPOSED	**39.76 dB/0.944/0.991**	**37.38 dB/0.932/0.989**	**37.82 dB/0.937/0.982**

**Table 4:** Numerical noisy image comparison of super-resolved chest X-ray images, up-scaled by a factor of 4×. The PSNR, SSIM, and MSIM are presented with bolded values to indicate the best performance.

Scale	Methods	CXR1	CXR2	CXR3
Noise		*PSNR/SSIM/MSIM*	*PSNR/SSIM/MSIM*	*PSNR/SSIM/MSIM*
X4 S&P 0.005	BICUBIC [24]	20.60 dB/0.606/0.628	19.23 dB/0.574/0.609	19.20 dB/0.552/0.687
	SRCNN [18]	21.90 dB/0.670/0.723	22.35 dB/0.652/0.701	21.90 dB/0.572/0.680
	VDSR [14]	23.12 dB/0.691/0.741	26.52 dB/0.684/0.719	23.12 dB/0.590/0.740
	EDSR [16]	31.43 dB/0.708/0.791	31.86 dB/0.701/0.881	**31.47 dB**/0.797/0.807
	RDN [17]	32.39 dB/0.797/0.890	32.45 dB/**0.827**/0.894	32.39 dB/0.806/0.893
	RCAN [8]	32.21 dB/0.798/0.842	32.42 dB/0.801/0.870	32.21 dB/0.798/0.842
	SNSRGAN [5]	31.67 dB/0.7944/0.890	29.33 dB/0.802/0.8903	31.67 dB/0.794/0.890
	PROPOSED	**32.43 dB/0.806/0.893**	**32.57 dB**/0.818/**0.892**	32.43 dB/**0.8008/0.8916**
X4 S&P 0.01	BICUBIC [24] SRCNN [18]	7.23 dB/0.013/0.011	7.18 dB/0.011/0.012	7.13 dB/0.010/0.011
		10.80 dB/0.026/0.034	9.08 dB/0.013/0.022	10.03 dB/0.042/0.023
	VDSR [14]	11.75 dB/0.045/0.047	12.28 dB/0.035/0.033	14.23 dB/0.045/0.054
	EDSR [16]	19.43 dB/0.22/0.15	17.28 dB/0.19/0.13	18.39 dB/0.170/0.14
	RDN [17]	20.47 dB/0.17/0.230	19.27 dB/0.21/0.17	19.23 dB/0.193/0.191
	RCAN [8]	20.04 dB/0.28/0.207	18.12 dB/0.19/0.16	18.17 dB/0.174/0.148
	SNSRGAN [5]	15.18 dB/0.160/0.19	16.22 dB/0.12/0.19	16.22 dB/0.154/0.172
	PROPOSED	**21.07 dB/0.305/0.2055**	**20.13 dB0.221/0.197**	**20.04 dB/0.217/0.195**
X4 S&P 0.02	BICUBIC [24] SRCNN [18]	6.98 dB/0.011/0.010	6.83 dB/0.0092/0.011	6.62 dB/0.010/0.0091
		10.45 dB/0.02/0.028	7.3 dB/0.011/0.019	8.7 dB/0.021/0.019
	VDSR [14]	11.80 dB/0.036/0.044	10.27 dB/0.028/0.056	10.43 dB/0.039/0.047
	EDSR [16]	19.33 dB/0.263/0.1804	16.95 dB/0.168/0.140	18.02 dB/0.151/0.137
	RDN [17]	20.06 dB/0.27/0.20	**19.15 dB/0.192/0.197**	18.97 dB/0.173/0.17
	RCAN [8]	19.65 dB/0.24/0.18	17.23 dB/0.171/0.12	17.15 dB/0.161/0.132
	SNSRGAN [5]	14.20 dB/0.13/0.15	7.3 dB/0.011/0.019	15.83 dB/0.142/0.157
	PROPOSED	**22.04 dB/0.260/0.178**	15.23 dB/0.175/0.12	**19.04 dB/0.198/0.175**

**Table 5:** Numerical evaluation (PSNR/SSIM) of four distinct models, all featuring an identical number of residual groups and blocks. The outcomes of the suggested model are bolded. ✓ include, ✗ not include for each RB in CA, concatenation and skip connection.

Our Network		RB			CXR 1 [29]	CXR 2 [30]	CXR 3 [30]
RG = 4, RB = 4 f = 64		**CA**	**Concatenation**	**Skip** **Connection**	**PSNR/SSIM**	**PSNR/SSIM**	**PSNR/SSIM**
	1	✓	✓	✓	39.69 dB/0.94	37.52 dB/0.9102	37.57 dB/0.93
	2	✗	✓	✓	**39.76 dB/0.944**	**37.79 dB/0.916**	**37.83 dB/0.93**
	3	✗	✗	✓	39.18 dB/0.948	37.83 dB/0.917	37.43 dB/0.937
	4	✓	✗	✓	38.98 dB/0.942	37.41 dB/0.902	37.14 dB/0.935
**RCAN [8]**		**RB**			**CXR 1**	**CXR 2**	**CXR 3**
RG = 4, RB = 4 f = 64		**CA**	**Concatenation**	**Skip** **connection**	**PSNR/SSIM**	**PSNR/SSIM**	**PSNR/SSIM**
	5	✓	✗	✓	39.55 dB/0.947	36.92 dB/0.927	37.77 dB/0.931
	6	✗	✗	✓	39.67 dB/0.94	37.7 dB/0.93	37.59 dB/0.93

## Data Availability

The datasets used in this paper are public datasets.
